# EEG Functional Connectivity and Cognitive Variables in Premanifest and Manifest Huntington’s Disease: EEG Low-Resolution Brain Electromagnetic Tomography (LORETA) Study

**DOI:** 10.3389/fphys.2020.612325

**Published:** 2020-12-17

**Authors:** Marianna Delussi, Virgilio Nazzaro, Katia Ricci, Marina de Tommaso

**Affiliations:** Applied Neurophyiology and Pain Unit-AnpLab-SMBNOS Department, Bari Aldo Moro University, Bari, Italy

**Keywords:** Huntington disease, EEG, functional connectivity, resting state networks, LORETA

## Abstract

**Background:**

Scientific literature does not offer sufficient data on electroencephalography (EEG) functional connectivity and its correlations with clinical and cognitive features in premanifest and manifest HD.

**Aim:**

This study tries to identify abnormal EEG patterns of functional connectivity, in conditions of “brain resting state” and correlations with motor decline and cognitive variable in Huntington’s disease (HD), in premanifest and manifest phase, looking for a reliable marker measuring disease progression.

**Method:**

This was an observational cross-sectional study; 105 subjects with age ≥18 years submitted to HD genetic test. Each subject underwent a neurological, psychiatric, and cognitive assessment, EEG recording and genetic investigation for detecting the expansion of the CAG trait. EEG connectivity analysis was performed by means of exact Low Resolution Electric Tomography (eLORETA) in 18 premanifest HD (pHD), 49 manifest HD (mHD), and 38 control (C) subjects.

**Results:**

HD patients showed a Power Spectral Density reduced in the alpha range and increased in delta band compared to controls; no difference was detectable between pHD and mHD; the Global Connectivity in pHD revealed no significant differences if compared to mHD. The Current Source Density was similar among groups. No statistically significant results when comparing pHD with C group, even in comparison of mHD with Controls, and pHD with mHD. mHD compared to Controls showed a significant increase in delta, alpha1, alpha2, beta2, and beta3. Lagged Phase Synchronization in delta, alpha1, alpha2, beta2, and beta3 bands was increased in HD compared to controls (*t* = −3.921, *p* < 0.05). A significant correlation was found in Regression Analysis: statistically significant results in pHD for the “Symbol Digit Modality Test and lagged phase synchronization” in the Beta1 (*r* = −0.806, *p* < 0.05) in the prefrontal regions. The same correlation was found in mHD for the Stroop Word Reading Test (SWRT) in the Alpha2 band (*r* = −0.759, *p* < 0.05).

**Conclusion:**

Increased phase synchronization in main bands characterized EEG in HD patients, as compared to controls. pHD were not dissimilar from mHD as regard to this EEG pattern. Increased phase synchronization correlated to cognitive decline in HD patients, with a similar trend in pHD, suggesting that it would be a potential biomarker of early phenotypical expression.

## Introduction

Huntington’s Disease (HD) is an inherited autosomal-dominant, progressive neurodegenerative disorder with phenotypic expression consisting of invalidating motor, cognitive, and psychiatric symptoms. It’s kinked to the progressive dysfunction and neuronal death in corticostriatal circuits (Novak and Tabrizi, 2011). The genetic test provides for the identification of the causative gene (mutated huntingtin, HTT). HD is inherited in 50% of first-degree relatives. The disease’s onset is associated with the first appearance of chorea movements but the early cognitive or psychiatric impairment is often present in clinical practice, before HD diagnosis is done (Tabrizi et al., 2011). Testing the CAG replication it’s possible to predict age at onset (Rosenblatt et al., 2006), the early stage of neurodegeneration and pathophysiological changes is not evident in clinical practice (Henry and Mochel, 2012). Clinical and instrumental assessment of the presymptomatic stage may provide for a potential biomarkers, may improve the knowledge about the neuronal circuits which are affected by mutated HTT in presymptomatic phase. The clinical relevance of electrophysiological tests in HD patients has already been investigated (Lefaucheur et al., 2002). The most frequent electroencephalographic abnormality described in HD is an amplitude reduction or suppression of alpha activity (Streletz et al., 1990; Bylsma et al., 1994; de Tommaso et al., 2003; Bellotti et al., 2004). Few studies have focused attention on electroencephalographic anomalies in subjects affected by HD in the preclinical phase. Already over 20 years ago, the reduction of alpha activity was found in subjects suffering from HD (Streletz et al., 1990), and it was also shown that the reduction of the power in the alpha band and the increase of the power in the beta and delta band, on the frontal and temporal regions, correlated with the severity of cognitive and neurological impairment in HD subjects (Bylsma et al., 1994). In recent years, researchers have paid more attention to exploring the functional state of the brain of individuals with HD, re-evaluating the potential of electroencephalography (EEG) as a key, non-invasive and inexpensive neurophysiological investigation technique for dynamic analysis of changes in brain activity, in both physiological and pathological conditions. Studies performed using quantitative EEG signal analysis (Q-EEG) techniques, including power spectral analysis, have documented changes in brain electrical activity, in all frequency bands, in HD subjects, as summarized by Piano et al. (2017b). In a pilot study, a global increase in absolute power was found in the delta band, but contrary to other studies, also in the alpha band in subjects with HD compared to controls; a loss of the “antero-posterior gradient” of the relative power in both α and δ bands, and a direct correlation with motor, cognitive and functional decline and with the extent of CAG expansion was found (Hunter et al., 2010). Currently, few studies have analyzed EEG features in HD patients during the preclinical phase in order to evaluate the relevance of EEG anomalies as potential biomarkers of phenotypic expression and clinical evolution. The employment of advanced analysis of EEG datasets, using Artificial Neural Network, allowed the detection of reduced alpha activity even in the EEG of preclinical mutation carriers for HD (de Tommaso et al., 2003). A more recent study performed on pre-symptomatic subjects, showed a reduced relative power in a narrow theta-alpha frequency band (7–9 Hz), a positive correlation between the relative power in the delta and theta band and the extent of the CAG expansion and an inverse correlation between the relative power in the alpha band and the extent of the CAG expansion, in subjects with HD in the preclinical phase, compared to controls (Ponomareva et al., 2014). The HD progression, its neurological and cognitive impairment, is slow and general categorization of EEG aberrations does not reach a sufficient sensitivity for the detection and localization of abnormalities. EEG tomography techniques such as LOw REsolution Tomography (eLORETA) have been developed in order to identify brain regions involved in neuropsychiatric disorders (Pascual-Marqui et al., 1994, 1999, 2002). The EEG functional connectivity analysis allows a detection of dysfunction that is more sensitive than that provided by the common EEG. LORETA computes a unique three dimensional electrical source distribution by assuming that the smoothest of all possible inverse solutions is the most plausible, which is consistent with the assumption that neighboring neurons are simultaneously and synchronously active (Pascual-Marqui et al., 1994, 1999). In LORETA the solution space is restricted to cortical gray matter and the hippocampus, as determined in the digitized Probability Atlas (Brain Imaging Center, Montreal Neurological Institute) based on the Talairach human brain atlas. Numerous studies provide validation for LORETA (Yao and He, 2001; Pascual-Marqui et al., 2002; Phillips et al., 2002a,b). Thus, LORETA, now widely accepted, low-cost and non-invasive diagnostic tool, combines the high time resolution of the EEG with a source localization method that permits three-dimensional tomography and functional connectivity analysis of brain electrical activity. LORETA has been applied to pre symptomatic genetic carriers in wake and sleep EEG in a cohort of 23 HD patients, analyzing cortical sources by eLORETA; the author found an increase of delta representation in the bilateral motor cortex (Piano et al., 2017b). In a following study, authors studied EEG functional connectivity in the same HD cohort, using lagged phase synchronization algorithm provided by eLORETA software, and confirmed changes in delta rhythm synchronization localized in motor areas (Piano et al., 2017a). de Tommaso et al. (2007) examined the contingent negative variation in 14 mildly demented HD patients, and Beste et al. (2008) investigated executive functions related to response inhibition in 13 HD patients by using event-related potentials and LORETA.

The aim of this study was to identify abnormal EEG patterns of functional connectivity in conditions of “brain resting state” and to find correlations with motor decline and cognitive variable, by using LORETA, in subjects affected by HD and in pre-symptomatic subjects, to look for possible pathological evidences and biomarkers, important for an early diagnosis, as well as to monitor the progression of the disease.

## Materials and Methods

### Study Design and Subject

This was an observational cross-sectional study, which was carried out at the Apulian Referral Center for HD between January 2014 and December 2018. We enrolled 116 consecutive non-medicated subjects, who came for the first time to our HD Center for admission to Daily Hospitalization for genetic and clinical investigation. The inclusion criteria taken into account for the present study were: age ≥18 years and the absence of previous treatment, first degree inheritance for HD, whereas the exclusion criteria included: presence of choreiform movements that affected the EEG recording, a past or ongoing history of medication, coexistence with other neurological and psychiatric conditions. According to the latter, 11 subjects were excluded from the study. The study sample thus consisted of a total of 105 subjects (50 m/55 f, *M* = 45.64 y, *SD* = 14.94). Each enrolled subject underwent a daily clinical evaluation, as reported below. Other diagnostic tests (biochemical and technical tests of neuroimaging including CT/MRI) useful for the differential diagnosis were also performed, in order to rule out other neurological and psychiatric conditions. Following the clinical-instrumental assessment and the genetic investigation, initially the study sample were divided into three groups, as detailed in Table 1. The Ethical Committee of Bari Policlinico General Hospital approved the study and each subject signed an informed consent.

**TABLE 1:** Demographic and clinical features of the 105 subjects included in the study sample.

	**All subjects**	**Controls**	**PreHD**	**HD**
Subjects (n)	105	38	18	49
Sex (Male/Female)	50/55	23/15	3/15	24/25
Age (years), mean (SD)	45.64 (14.94)	41.95 (15.76)	37 (12.46)	51.61 (12.77)
Duration of disease (years), mean (SD)				3.75 (3.13)
UHDRS, mean (SD)	17.11 (21.02)	0.52 (0.87)	2.2 (2.14)	34.83 (18.16)
TFC, mean (SD)	10.80 (3.59)	13 (0)	13 (0)	8.41 (4.01)
Function: -Assessment Score, mean (SD) -Score Incomplete, mean (SD) -Independence in%, mean (SD)	21.26 (5.84) 21.26 (5.84) 89.38 (17.56)	25 (0) 25 (0) 100 (0)	25 (0) 25 (0) 100 (0)	17.31 (6.31) 17.31 (6.31) 78.14 (19.78)
SDMT: -Correct, mean (SD) -Errors, mean (SD)	32.03 (21.58) 0.43 (1.20)	44.81 (15.09) 0.08 (0.39)	45.55 (17.25) 0 (0)	13.54 (14.24) 0.96 (1.71)
VFT-Total Correct, mean (SD)	17.97 (10.78)	26.5 (8.71)	23.64 (6.00)	9.09 (5.34)
STROOP Color Naming: -Correct, mean (SD) -Errors, mean (SD) -Self-corrected, mean (SD)	58.38 (22.70) 0.23 (0.73) 0.46 (0.80)	76.62 (12.46) 0.08 (0.27) 0.35 (0.80)	67.82 (12.16) 0.64 (1.21) 0.82 (0.87)	40.31 (17.60) 0.22 (0.75) 0.44 (0.76)
STROOP Word Reading: -Correct, mean (SD) -Errors, mean (SD) -Self-corrected, mean (SD)	64.96 (25.93) 0.28 (1.37) 0.21 (0.66)	84.38 (14.32) 0.04 (0.20) 0.15 (0.46)	81 (13.01) 0.09 (0.30) 0 (0)	42.97 (18.79) 0.55 (2.00) 0.32 (0.87)
STROOP Interference: -Correct, mean (SD) -Errors, mean (SD) -Self-corrected, mean (SD)	34.48 (16.43) 1.13 (2.59) 1.39 (2.11)	44.68 (9.43) 0.76 (2.55) 0.72 (0.94)	46.55 (8.27) 0.45 (1.04) 0.73 (0.90)	18.33 (10.74) 1.83 (2.35) 2.36 (2.87)
PBAs: -Depression, mean (SD) -Irritability/Aggressiveness, mean (SD) -Psychosis, mean (SD) -Apathy, mean (SD) -Executive Function, mean (SD)	4.24 (5.91) 1.97 (3.84) 0.28 (1.26) 0.86 (2.16) 0.51 (1.90)	1.73 (3.29) 1.12 (2.69) 0 (0) 0.19 (0.80) 0.04 (0.20)	1.2 (2.04) 0.1 (0.32) 0 (0) 0 (0) 0 (0)	6.97 (6.90) 3.14 (4.69) 0.57 (1.75) 1.6 (2.83) 1 (2.63)
MMSE, mean (SD)	25.91 (4.70)	29.57 (0.85)	30 (0)	23.70 (4.67)

### Clinical Evaluation

#### Neurological and Psychiatric Assessment

We performed the Diagnostic Confidence Level (DCL) of the Total Motor Score (TMS) as part of the Huntington’s Disease Rating Scale (UHDRS) (Huntington Study Group, 1996; Hogarth et al., 2005) and the Total Functional Capacity Scale (TFC) (Shoulson, 1981) in order to assess the presence of motor manifestations, which were clinically interpreted as “unequivocal signs of HD,” and the PBA-s (Kingma et al., 2008) for the psychiatric assessment.

#### Cognitive Assessment

Mini-Mental State Examination (MMSE) (Folstein et al., 1975), Symbol Digit Modality Test (SDMT) (Smith, 2007), Categorical Verbal Fluency (FAS) (Tombaugh et al., 1999), Stroop Test (ST) (Stroop, 1935) were administered.

#### Genetic Investigation

The genetic test was performed on peripheral blood lymphocytes in order to define the condition of certain carrier by detecting the expansion of the CAG trait 40 in an allele of the IT-15 gene.

#### Electroencephalographic Examination

The electroencephalographic (EEG) recording was performed with the subject at rest, positioned in a quiet room with an ambient temperature of 21–23°C, in an awake and relaxed state, in a sitting position, in a softly lit and soundproofed environment. The EEG recordings were obtained by placing 61 surface electrodes on the scalp, according to an extension of the International System 10–20 (Fp1, Fpz, Fp2, F7, F3, Fz, F4, F8, T3, C3, Cz, C4, T4, T5, P3, Pz, P4, T6, O1, Oz, O2, FC2, FC1, CP1, CP2, PO3, PO4, FC6, FC5, CP5, CP6, AF7, AF3, AFz, AF4, AF8, F5, F1, F2, F6, FT7, FC3, FCz, FC4, FT8, C5, C1, C2, C6, TP7, CP3, CPz, CP4, TP8, P5, P1, P2, P6, PO7, POz, PO8), by the use of a pre-wired headset, in which each electrode is referred to a common reference electrode positioned on the nasion (lead in common reference inactive), also applying two electrodes to detect eye movements (electro-oculographic channel or EOG) and an earth electrode on the back of the hand. The electrode impedances were kept below 5 kΩ and a sampling frequency of 256 Hz was used for the acquisition. The EEG signals were amplified, filtered, and saved on a biopotential analyzer (Micromed System Plus, Micromed, Mogliano Veneto, Italy). After 2 min of adaptation, we asked to subjects to remain relaxed for 3 min with eyes closed while EEG was recorded.

### Data Analysis

Preprocessing was performed in MATLAB using the EEGLAB 14_1_1 tool. The data were first high-pass filtered at 1 Hz to remove slow drifts, with 70 Hz as low pass filter. Next, a notch filter at 50 Hz (L: 48, H: 52) was applied to remove power line noise artifacts. A preliminary visual inspection allowed to delete EEG segments affected by hyperkinetic movements, as indicated by the technician. Artifact components were then automatically removed considering 150 uV as critical value of amplitude and the components recorded on the electrooculogram (EOG) channels. In fact, blinking was present even in closed eyes conditions, especially in HD patients. Bad channels were identified by a semiautomatic method based on visual detection and channel statistics. To precompute channel measures, spherical interpolation of missing channels and deletion of Independent Component Analysis (ICA) artifact components pre-tagged in each dataset was performed. Channels presenting with distributions of potential values further away from the Gaussian distribution than other scalp channels were also removed. 60 s of artifact free EEG were selected in each case for the analysis reported below.

### EEG Frequency Analysis

Electroencephalography frequency analysis was computed in Matlab using the spectopo parameters included into the EEGLAB 14_1_1 tool, with the computation of power spectral density [log_10_ (μV^2^/Hz)].

### EEG Signal Source Analysis

The source analysis of the EEG signal (EEG rhythms), representative of the cortical distribution of the “current source density” starting from the EEG data recorded by surface electrodes, was performed using the last version of the LORETA software (Yao and He, 2001; Pascual-Marqui et al., 2002; Phillips et al., 2002a,b). LORETA uses a realistic head model and EEG electrode coordinates, based on the digitized atlas Talairach, provided by the Brain Imaging Center of the Montreal Neurological Institute (Mazziotta et al., 2001); the 3D solution space is limited to the cortical gray matter, divided into 6,239 voxels, with a spatial resolution of 5 mm^3^, within which it is possible to identify various cortical macro-regions of interest (ROIs), each of which can enclose different Brodmann areas (BA). For the present study, 61 electrode coordinates were created, starting from the 61 registering surface electrodes, on the basis of which an average head model was interpolated, necessary for the calculation of the “transformation matrix,” for the conversion of the electrical potential differences recorded at the scalp level into “current density.” The epochs of resting state – artifact free – EEG (rsEEG),were converted and imported into LORETA, to first create the “EEG cross-spectra” and subsequently, to calculate and elaborate the corresponding functional images of cortical distribution of the generators of oscillatory electrical activity in different frequency bands: delta (2–4 Hz), theta (4–8 Hz), alpha 1 (8–10 Hz), alpha 2 (10–13 Hz), beta 1 (13–16 Hz), beta 2 (16–20 Hz), and beta 3 (20–30 Hz).

### Functional Connectivity Analysis

The LORETA software was also used to analyze functional connectivity using a “voxel-wise” approach for the determination of the ROIs, created on the basis of the coordinates of the cortical voxels and centered, each one in respect to the coordinates of a given voxel, corresponding to the predetermined “ROI centroid.” Thus 61 ROIs were created, using as ROI centroids the coordinates of the voxels, at the level of which the highest levels of electrical activity were found in the various groups and in the various frequency bands. For the definition of the extension of each ROI, the “single nearest voxel” option was chosen (Table 2). As for the analysis of functional connectivity, for each pair of ROIs a new non-linear method was used, the “lagged phase synchronization,” which measures the “similarity” (correct phase synchronization value) between signals in the frequency domain based on the “normalized Fourier transforms,” which, following the breakdown of total connectivity into an instantaneous and a delayed component (“lagged”), eliminates the artifactual instantaneous component (low spatial resolution and conduction volume) responsible for “polluted” connectivity patterns and provides a more precise estimate of functional connectivity (Pascual-Marqui et al., 2011). Finally, the association between functional connectivity and decline in motor, cognitive, and psychiatric features was analyzed in pHD (premanifest HD) and mHD (manifest HD) subjects.

**TABLE 2:** The 61 ROIs created in LORETA for the analysis of functional connectivity in terms of “lagged phase synchronization.”

**Brodmann area (BA) Anatomical region Lobe**	**ROI centroid coordinates X Y Z**
BA 10 Superior Frontal Gyrus Frontal lobe	−25, 65, −5
BA 10 Medial Frontal Gyrus Frontal lobe	5, 65, −5
BA 10 Superior Frontal Gyrus Frontal lobe	25, 65, −5
BA 47 Inferior Frontal Gyrus Frontal lobe	−50, 40, −10
BA 10 Middle Frontal Gyrus Frontal lobe	−40, 45, 30
BA 8 Superior Frontal Gyrus Frontal lobe	5, 45, 50
BA 10 Middle Frontal Gyrus Frontal lobe	40, 45, 30
BA 47 Inferior Frontal Gyrus Frontal Lobe	50, 40, −10
BA 21 Middle Temporal Gyrus Temporal lobe	−65, −15, −15
BA 3 Postcentral Gyrus Parietal lobe	−50, −20, 60
BA 6 Superior Frontal Gyrus Frontal lobe	5, −10, 70
BA 1 Postcentral Gyrus Parietal lobe	55, −20, 55
BA 21 Middle Temporal Gyrus Temporal lobe	70, −20, −10
BA 37 Inferior Temporal Gyrus Temporal lobe	−60, −65, −10
BA 7 Inferior Parietal Lobule Parietal lobe	−40, −70, 45
BA 7 Precuneus Parietal lobe	−5, −65, 65
BA 7 Inferior Parietal Lobule Parietal lobe	45, −70, 45
BA 37 Middle Occipital Gyrus Occipital lobe	55, −70, 0
BA 19 Middle Occipital Gyrus Occipital lobe	−20, −100, 10
BA 18 Cuneus Occipital lobe	−5, −100, 15
BA 18 Middle Occipital Gyrus Occipital lobe	20, −100, 5
BA 6 Middle Frontal Gyrus Frontal lobe	30, 10, 65
BA 6 Superior Frontal Gyrus Frontal lobe	−20, 15, 65
BA 5 Postcentral Gyrus Parietal lobe	−30, −45, 70
BA 5 Postcentral Gyrus Parietal Lobe	30, −45, 70
BA 19 Cuneus Occipital lobe	−30, −90, 30
BA 19 Precuneus Parietal lobe	35, −85, 35
BA 44 Inferior Frontal Gyrus Frontal lobe	60, 15, 20
BA 45 Inferior Frontal Gyrus Frontal lobe	−60, 15, 20
BA 40 Supramarginal Gyrus Parietal lobe	−65, −45, 30
BA 40 Supramarginal Gyrus Parietal lobe	65, −50, 30
BA 11 Middle Frontal Gyrus Frontal lobe	−40, 55, −10
BA 10 Superior Frontal Gyrus Frontal lobe	−25, 60, 20
BA 10 Superior Frontal Gyrus Frontal lobe	5, 60, 30
BA 10 Middle Frontal Gyrus Frontal lobe	30, 60, 15
BA 10 Middle Frontal Gyrus Frontal lobe	45, 55, −5
BA 45 Inferior Frontal Gyrus Frontal lobe	−55, 35, 5
BA 8 Superior Frontal Gyrus Frontal lobe	−20, 45, 45
BA 8 Superior Frontal Gyrus Frontal lobe	15, 45, 50
BA 46 Middle Frontal Gyrus Frontal lobe	50, 45, 10
BA 21 Middle Temporal Gyrus Temporal lobe	−60, 0, −15
BA 8 Middle Frontal Gyrus Frontal lobe	−45, 15, 50
BA 6 Superior Frontal Gyrus Frontal lobe	5, 20, 65
BA 8 Middle Frontal Gyrus Frontal lobe	5, 15, 45
BA 21 Middle Temporal Gyrus Temporal Lobe	60, 5, −15
BA 3 Postcentral Gyrus Parietal lobe	−65, −15, 30
BA 6 Precentral Gyrus Frontal lobe	−30, −15, 70
BA 6 Precentral Gyrus Frontal lobe	35, −15, 70
BA 3 Postcentral Gyrus Parietal lobe	65, −15, 30
BA 21 Middle Temporal Gyrus Temporal lobe	−65, −45, −10
BA 40 Inferior Parietal Lobule Parietal lobe	−50, −50, 55
BA 5 Postcentral Gyrus Parietal lobe	−5, −50, 70
BA 40 Inferior Parietal Lobule Parietal lobe	50, −50, 55
BA 21 Middle Temporal Gyrus Temporal lobe	70, −35, −10
BA 39 Middle Temporal Gyrus Temporal lobe	−55, −70, 25
BA 7 Superior Parietal Lobule Parietal lobe	−25, −65, 65
BA 7 Superior Parietal Lobule Parietal lobe	25, −65, 65
BA 39 Angular Gyrus Temporal lobe	50, −75, 30
BA 19 Middle Occipital Gyrus Occipital Lobe	−40, −90, 5
BA 19 Cuneus Occipital lobe	−5, −90, 35
BA 19 Middle Occipital Gyrus Occipital lobe	40, −90, 5

### Statistical Analysis

For power spectrum, we used statistical analysis provided by EEGLAB tool, which was the parametric statistic ANOVA test (mHD vs. pHD vs. C) with Bonferroni correction for multiple comparisons. For sLoreta current source density, the analysis was performed by means of the statistical non-parametric mapping methodology known as Fisher’s permutation test (Nichols and Holmes, 2002), integrated with Holmes’ non-parametric correction procedure for multiple comparisons (Holmes et al., 1996), both included in the LORETA software. As far as the measures of “current source density” are concerned, comparisons were made between pHD and control (C) subjects; mHD and C subjects; and mHD and pHD subjects. In this case, a “*t*-statistic on Log transformed data” test was chosen, with a variance smoothing parameter of 0 and a number of randomizations of 5,000. The test allowed to calculate the threshold values in terms of “log F-ratio” and yielded to a file containing the computed extremes of probability (ExtremePs), the corresponding maximal thresholds, and the thresholds at probability values of *p* < 0.01, *p* < 0.05, and *p* < 0.10, with *p* < 0.05 being indicative of statistical significance (Friston et al., 1991). The same group subdivision and the same comparisons were applied for the measurements of functional connectivity in terms of “lagged phase synchronization.” For this purpose, a “*t*-statistic” test was carried out, again with a variance smoothing parameter of 0 and 5,000 randomizations, and again a file was created containing the ExtremePs, the maximal thresholds and the thresholds at probability values of *p* < 0.01, *p* < 0.05, and *p* < 0.10. The LORETA software was also used to establish the correlations between the functional connectivity files in terms of “lagged phase synchronization” in the pHD and mHD groups individually and the variables assessed in clinical practice (Table 1). A regression analysis with 5,000 randomizations was made in order to calculate the Pearson’s coefficient “r” to define both the maximal thresholds and the thresholds at probability values of *p* < 0.01, *p* < 0.05, and *p* < 0.10, and the corresponding ExtremePs.

## Results

**FIGURE 1:**
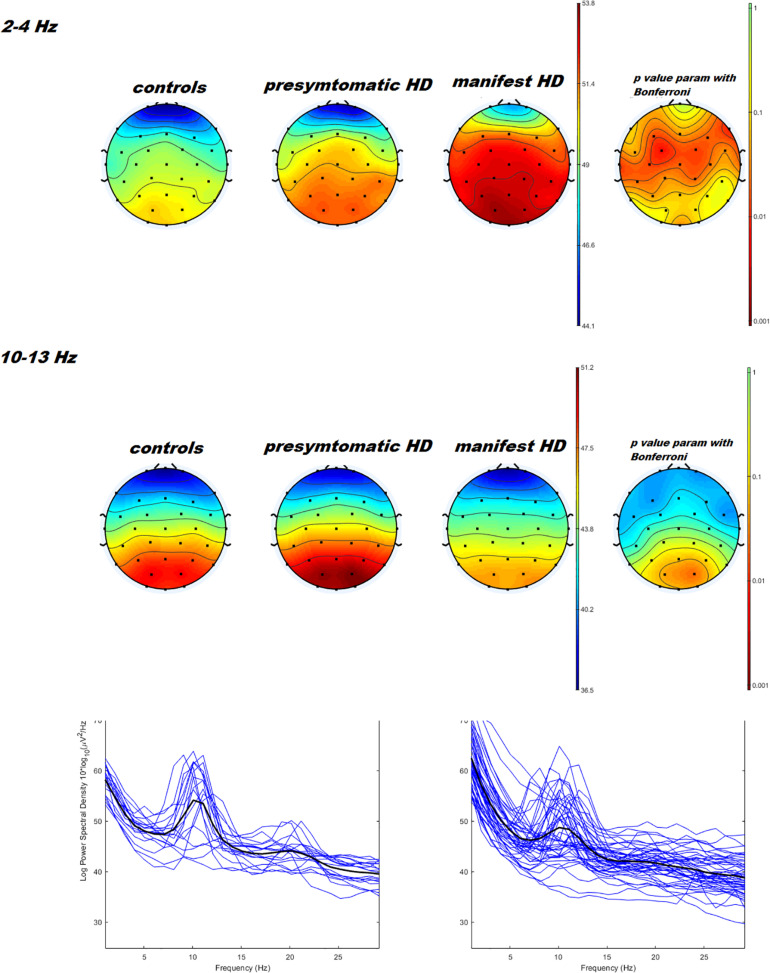
Topographical maps and statistical analysis of power density in delta e alpha band. On the left side, results of three ways ANOVA with controls vs. pHD vs. mHD is reported. *P* values < 0.05 are represented in orange and red colors. The two ways ANOVA test comparing pHD vs. mHD and pHD vs. controls was not significant. At the bottom of the figure, example of power spectra averaged across O1, O2, and Oz channels in pHD and mHD are reported.

**FIGURE 2:**
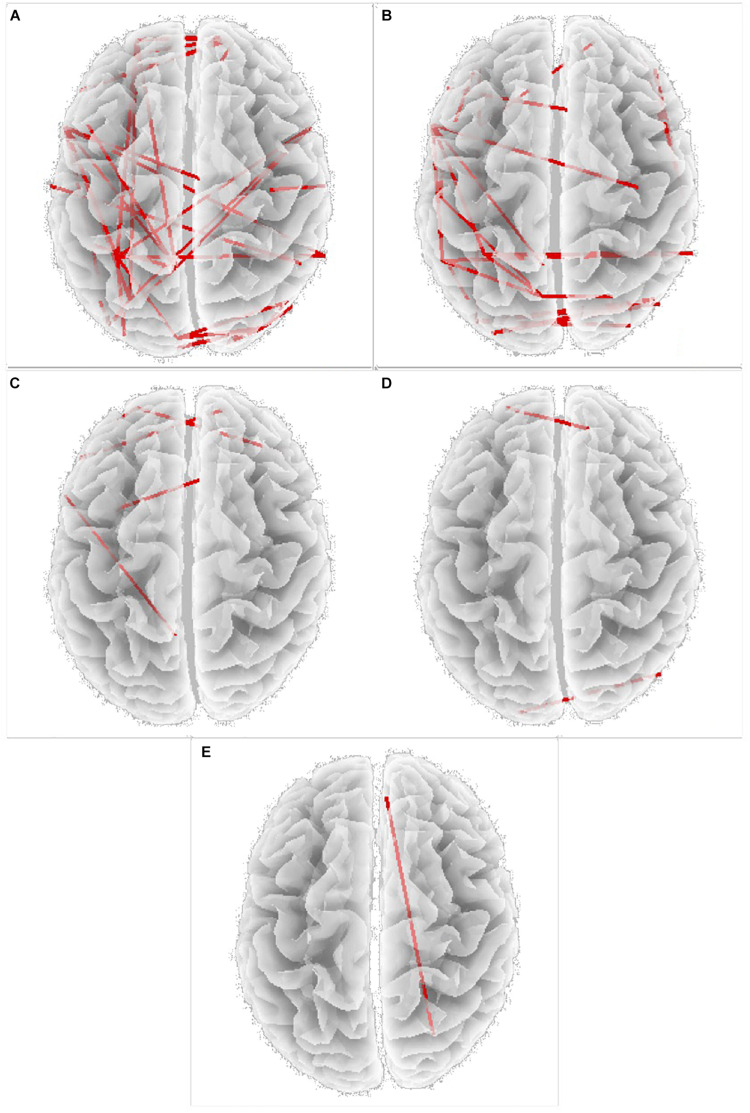
Increase in “lagged phase synchronization” (red lines) in **(A)** Delta band; **(B)** Alpha 1 band; **(C)** Alpha 2 band; **(D)** Beta 2 band; **(E)** Beta 3 band, Specific BAs involved are reported in Table 3.

**FIGURE 3:**
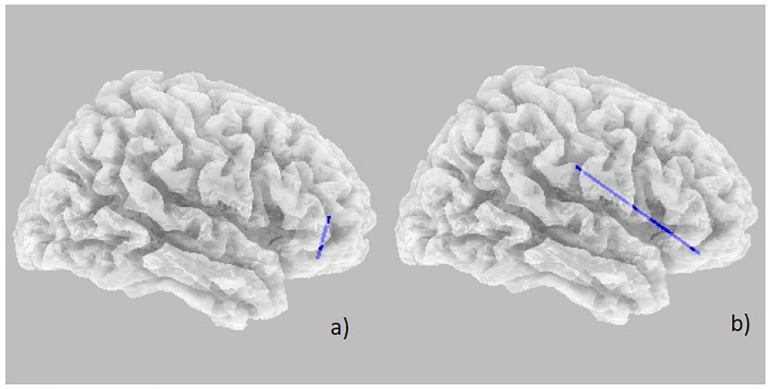
Decrease in “lagged phase synchronization” (blue lines). **(a)** Correlations with SDMT (correct answer item) in pHD in the Beta 1 band between right BA 46 and right BA 47; **(b)** Correlation with SWRT (error item) in mHD patients in the Alpha 2 band between right BA 47 and right BA 3.

Power spectral density was reduced in the alpha range and increased in delta band in HD patients compared to controls (Figure 1). The pHD group was not significantly different from HD group (Figure 1). Analysis of “current source density” yielded to no statistically significant results when comparing pHD with C subjects, mHD with C subjects, and pHD with mHD subjects. Statistical significance was found in “lagged phase synchronization” when comparing mHD with C subjects; the threshold for significance was *t* = −3.921 (corresponding to *p*< 0.05), with significant modifications in delta, alpha1, alpha2, beta2, and beta3 bands. Compared to C, mHD subjects showed an increase in “lagged phase synchronization” in all of the above mentioned bands (Figures 2A–E and Table 3). In the comparisons between pHD and C subjects, and pHD and mHD subjects, no statistically significant results were found. In regression analysis, we found statistically significant results in pHD subjects for the SDMT (correct item) and in mHD subjects for the Stroop Word Reading Test (SWRT) (errors item), while in controls no relevant association between phase lagged synchronization and cognitive performance was found. In the pHD, the threshold of significance was *r* = −0.806 (corresponding to *p*< 0.05), with increased synchronization in Beta 1 band corresponding to a worse cognitive performance. The correlation was located in the right prefrontal regions (BA 46 corresponding to Middle Frontal Gyrus and the right BA 47 corresponding to Inferior Frontal Gyrus) (Figure 3a and Table 4). In the mHD, the threshold of significance was *r* = −0.759 (corresponding to *p*< 0.05), with a negative correlation in the Alpha2 band between the right BA 47 and BA 3 (corresponding Postcentral Gyrus, Parietal lobe) (Figure 3b and Table 5). We also performed a one way ANOVA model and the *post hoc* Bonferroni (*P*≤ 0.05) to check for SDMT and sub-items differences among pHD and mHD (*F* = 27.8, *P* = 0.012) and for SWRT and sub-items differences among pHD and mHD (*F* = 7.6, *P* = 0.018).

**TABLE 3:** Changes in “lagged phase synchronization” in mHD subjects compared to C subjects in all frequency bands and the involved BAs.

**Frequency bands**	**Areas of significance**
Delta	Increased LPS between: -Left BA 18 and right BA 19 -Left BA 19 and left BA 45; left BA 7; left BA 5 -Left BA 19 and left BA 5; right BA 7; right BA 19 -Left BA 7 and left BA 19; left BA 45; left BA 10; right BA 44 -Left BA 5 and left BA 19; left BA 45; left BA 10; right BA 44; right BA 40 -Left BA 5 and left BA 19; left BA 21; left BA 45; left BA 8; left BA 10; right BA 44 -Left BA 3 and right BA 40 -Left BA 45 and BA 19; left BA 7; left BA 5; right BA 6; right BA 40 -Left BA 10 and left BA 5; left BA 7; right BA 10 -Right BA 10 and left BA 10; left BA 11; left BA 45; left BA 47; right BA 8 -Right BA 44 and left BA 5; left BA 7 -Right BA 6 and right BA 3 -Right BA 40 and left BA 5; left BA 45 -Right BA 7 and left BA 19; right BA 19 -Right BA 19 and left BA 19; left BA 18; right BA 18; right BA 39 -Right BA 19 and left BA 18; right BA 7 -Right BA 18 and right BA 19; right BA 37
Theta	None
Alpha 1	Increased LPS between: -Left BA 19 and left BA 11; right BA 7; right BA 19; right BA 39 -Left BA 19 and right BA 7; right BA 39 -Left BA 7 and left BA 40; left BA 5; left BA 3; right BA 7 -Left BA 40 and left BA 45; right BA 40; left BA 7 -Left BA 5 and left BA 7; left BA 45; right BA 40 -Left BA 3 and left BA 7; left BA 21 -Left BA 45 and left BA 37; left BA 40; left BA 5; right BA 6 -Left BA 21 and left BA 7; left BA 21 -Left BA 21 and left BA 21; left BA 3; right BA 10 -Left BA 45 and right BA 6 -Left BA 11 and left BA 19; left BA 8 -Right BA 46 and right BA 21 -Right BA 21 and right BA 46; right BA 47; right BA 10; right BA 8 -Right BA 40 and left BA 40; left BA 5 -Right BA 7 and left BA 19 -Right BA 39 and left BA 19
Alpha 2	Increased LPS between: -Left BA 5 and left BA 45 -Left BA 45 and right BA 10 -Left BA 21 and right BA 6 -Left BA 10 and left BA 11; right BA 47
Beta 1	None
Beta 2	Increased LPS between: -Left BA 19 and right BA 39 -Left BA 10 and right BA 8
Beta 3	Increased LPS between: -Right BA 8 and right BA 7

**TABLE 4:** Changes in “lagged phase synchronization” in pHD subjects following correlation with SDMT (correct answers item) in all frequency bands and the involved BAs.

**Frequency bands**	**Areas of significance**
Delta	None
Theta	None
Alfa 1	None
Alfa 2	None
Beta 1	Decreased LPS between: -Right BA 46 and right BA 47
Beta 2	None
Beta 3	None

**TABLE 5:** Changes in “lagged phase synchronization” in mHD subjects following correlation with SWRT (errors item) in all frequency bands and the involved BAs.

**Frequency bands**	**Areas of significance**
Delta	None
Theta	None
Alpha 1	None
Alpha 2	Decreased LPS between: -Right BA 47 and right BA 3
Beta 1	None
Beta 2	None
Beta 3	None

## Discussion

This was an observational cross-sectional study based on the analysis of the rsEEG functional connectivity index in pHD patients compared with mHD and C group by means of EEG LORETA. We tried to investigate if rsEEG functional connectivity could provide a quantification method for possible early detections of subcortical dysfunction occurring prior to or concomitant with motor or cognitive disturbances in HD. As the solution space of LORETA is restricted to cortical gray matter and the hippocampus, we were primarily interested in the utility of LORETA in a subcortical disease such as HD. EEG spectral analysis confirmed reduced alpha rhythm and increased delta rhythms expression in mHD. In accord with previous studies (Bylsma et al., 1994; de Tommaso et al., 2003), pHD patients were not dissimilar from mHD, as well as from controls, as subtle brain changes occurring during the premanifest stage, could yield to an intermediate EEG phenotype. The origin of the alpha rhythm is still unclear but, in literature, the abnormality it’s associated with a primary dysfunction affecting the cortex (Lopes da Silva et al., 1980) or with a dysfunction of subcortical structures which modulate cortical activity, mainly the thalamus. The pathophysiological background in HD is the bilateral striatal atrophy, which leads to a disruption of the cortico-striato-thalamocortical circuits, causing a decrease in thalamic alpha activity (Hughes and Crunelli, 2005; Alper et al., 2006). Therefore, the results can be interpreted as an effect of abnormal subcortical modulation of the alpha rhythm due to the dysfunctional action of the thalamus on the cortical activities (Hughes and Crunelli, 2005; Alper et al., 2006). The observed increase in delta power in HD is present in the scientific literature (Bylsma et al., 1994) but alpha rhythm suppression seemed to better discriminate HD carriers from controls (de Tommaso et al., 2003). The analysis of current source density does not allow to separate pHD gene carriers from mHD and healthy controls, though different changes under the statistical significant threshold were detectable. This result could be in apparent contradiction to the power spectra density changes reported above, but it would indicate that the fundamental cortical sources of main rsEEG rhythms were not different from controls in mHD. Moreover, event related activity showed different cortical sources in mHD as compared to controls (de Tommaso et al., 2007). In a previous study, delta activity increase in HD was identified in motor areas (Piano et al., 2017b). Our HD series confirmed a bilateral frontal distribution with topographical distribution of delta activity increase, coherent with bilateral motor regions, though LORETA source analysis remained below the statistical significance. Changes in phase synchronization emerged when comparing mHD with C group. Increased phase synchronization involved delta, alpha1, alpha2, beta2, and beta3 bands. This analysis allowed to separate mHD from C subjects with good accuracy and precision. Disruption in the dynamical properties orchestrating local firing rates and global network oscillation changes are observed in neurodegenerative disorders, according to a unifying “oscillopathy” concepts (Nimmrich et al., 2015). This is particularly well illustrated in the basal ganglia (BG) functioning in Parkinson’s disease, where the loss of dopamine is associated with the abnormal oscillatory synchronization among and between basal ganglia nuclei; the synchronization abnormality primarily involves the beta rhythm (Crompe et al., 2020; Liu et al., 2020). In HD, neurodegeneration affects the inter-connectivity of striatal medium-sized spiny neurons (MSNs) (Tabrizi et al., 2009), and disrupted pattern of cortical oscillations induced by subthalamic nuclei were described in animal models (Callahan and Abercrombie, 2015). A decline in phase locking was also observed in PreHD patients during a cognitive “NoGo” task (Beste et al., 2011). Our results indicate a clear modification of resting state EEG (rsEEG) synchronization in mHD, while pHD confirmed an intermediate bioelectrical phenotype, with a lack of significant changes either in comparison to mHD or controls. The increased synchronization was clearly present in the most of considered bands, confirming to be a generalized phenomenon of altered cortical networks oscillation, probably due to the striatal degeneration and change in interconnectivity and synaptic connection efficiency with cortical regions (Tabrizi et al., 2009).

Increased synchronization in delta band involved several cortical regions, including the premotor area, according to previous studies (Piano et al., 2017a). We did not find a correlation with motor impairment, rather increased synchronization corresponded to worse cognitive performance in both mHD and pHD, in well localized BA areas in prefrontal regions. SDMT and SWRT are both relevant clinical markers in HD research and disease progression (Martinez-Horta et al., 2020). Considering that HD is characterized by an accumulation of subcortical and cortical dysfunctions with a disruption of cortico-subcortical circuits, the current neuroimaging findings are not able to detect in which exact area of the cerebral cortex meaningful neuronal loss firstly occurs. However, the frontal cortex and the basal ganglia are mainly seen as a functional unit in HD, with a possible disruption of a cortico-subcortical and cortico-cortical circuits (Painold et al., 2011). SDMT changes occur very early in the development of HD (Martinez-Horta et al., 2020). Our pHD subjects showed a quite normal SDMT performance, but subtle cognitive decline with worse scores, corresponded to a cortical dysfunction in the right prefrontal regions, consisting of increased synchronization in the fast EEG rhythms. In mHD, the SDMT was uniformly compromised among patients, while residual cognitive abilities in SWRT corresponded to a reduced abnormality in the pattern of alpha rhythm synchronization in the same prefrontal areas and connections with sensory motor regions. This was in accord with previous findings of disrupted connectivity between prefrontal and sensory motor regions in impaired processing speed (Krukow et al., 2018). The lack of correlation between phase lagged synchronization in main EEG bands and motor and functional impairment, as well as disease duration, could thus suggest that the abnormal modality of neural networks oscillations could vary among patients mainly in the cortical regions subtending cognitive impairment, as the prefrontal and dorsolateral prefrontal ones. Studies employing FMRI, confirmed altered connectivity between striatum and prefrontal regions in early and pHD gene carriers (Kronenburger et al., 2019), probably due to compensatory mechanisms occurring in those cortical regions subtending the complex sensory motor integration involved in the solution of cognitive tasks (Kronenburger et al., 2019).

## Study Limitation

The small sample size of pHD group is the main limitation of this study. The unbalance of sample size between pHD, mHD and C groups could have negative impact on statistical analysis. Moreover, this limitation is frequent for rare genetic diseases.

## Conclusion

The results of this observational cross-sectional study show that hypersynchronization is a feature of rsEEG in mHD. The altered connectivity pattern in the prefrontal cortex could subtend the onset and development of cognitive dysfunction in HD genetic abnormality carriers. Innovative approaches to EEG functional connectivity in the broader context of network physiology, based on interaction pattern among different rhythms (Liu et al., 2015; Lin et al., 2020), could provide for a unified hypothesis of brain dysfunction as a hallmark of early phenotypical changes. Longitudinal multicenter study designs could clarify the possible predictive role of rsEEG hyper synchronization in disease onset and progression.

## Data Availability Statement

The raw data supporting the conclusions of this article will be made available by the authors, without undue reservation.

## Ethics Statement

The studies involving human participants were reviewed and approved by the Ethical Committee of Bari Policlinico General Hospital. The patients/participants provided their written informed consent to participate in this study.

## Author Contributions

MD: study design, interview preparation, patient selection, manuscript preparation, and study coordination. VN: manuscript preparation and data analysis. KR: neurophysiopathology laboratory technique and data analysis. MT: study coordination, manuscript preparation, data analysis, and manuscript editing. All authors contributed to the article and approved the submitted version.

## Conflict of Interest

The authors declare that the research was conducted in the absence of any commercial or financial relationships that could be construed as a potential conflict of interest.

## Abbreviations

HD: Huntington Disease
mHD: manifest HD
pHD: premanifest HD
C: Control group
MMSE: Mini-Mental State Examination
SDMT: Symbol Digit Modality Test
FAS: Categorical Verbal Fluency
ST: Stroop Test
SWRT: Stroop Word Reading Test
EEG: electroencephalography
ROIs: cortical macro-regions of interest
BA: Brodmann areas
rsEEG: resting state EEG
ExtremePs: extremes of probability.
